# Viral Specific Factors Contribute to Clinical Respiratory Syncytial Virus Disease Severity Differences in Infants

**DOI:** 10.4172/2327-5073.1000206

**Published:** 2015-06-29

**Authors:** Tonya M Thompson, Philippa L Roddam, Lisa M Harrison, Jody A Aitken, John P DeVincenzo

**Affiliations:** 1 University of Tennessee School of Medicine, Department of Pediatrics; 2 University of Tennessee Graduate School of Health Sciences, Department of Molecular Sciences; 3 Le Bonheur Children’s Hospital; 4 The Children’s Foundation Research Institute at Le Bonheur Children’s Hospital, Memphis, TN 38103; 5 University of Arkansas for Medical Sciences, Department of Pediatric Emergency Medicine, Little Rock, AR 72202

**Keywords:** Respiratory syncytial virus (RSV), Cytokines, Chemokines, Clinical isolates, Disease severity

## Abstract

**Background:**

There is a wide range of severity of respiratory syncytial viral (RSV) disease in previously healthy infants. Host factors have been well demonstrated to contribute to disease severity differences. However the possibility of disease severity differences being produced by factors intrinsic to the virus itself has rarely been studied.

**Methods:**

Low-passage isolates of RSV collected prospectively from infants with different degrees of RSV disease severity were evaluated *in vitro*, holding host factors constant, so as to assess whether isolates induced phenotypically different cytokine/chemokine concentrations in a human lung epithelial cell line. Sixty-seven RSV isolates from previously healthy infants (38 hospitalized for acute RSV infection (severe disease) and 29 never requiring hospitalization (mild disease)) were inoculated into A549, lung epithelial cells at precisely controlled, low multiplicity of infection to mimic natural infection. Cultures were evaluated at 48 hours, 60 hours, and 72 hours to evaluate area under the curve (AUC) cytokine/chemokine induction.

**Results:**

Cells infected with isolates from severely ill infants produced higher mean concentrations of all cytokine/chemokines tested (IL-1α, IL-6, IL-8 and RANTES) at all-time points tested. RSV isolates collected from infants with severe disease induced significantly higher AUCIL-8 and AUCRANTES secretion in infected cultures than mild disease isolates (p=0.028 and p=0.019 respectively). IL-8 and RANTES concentrations were 4 times higher at 48 hours for these severely ill infant isolates. Additionally, 38 isolates were evaluated at all-time points for quantity of virus. RSV concentration significantly correlated with both IL-8 and RANTES at all-time points. Neither cytokine/chemokine concentrations nor RSV concentrations were associated with RSV subgroup.

**Discussion:**

Infants’ RSV disease severity differences may be due in part to intrinsic viral strain-specific characteristics.

## Introduction

Respiratory syncytial virus (RSV) is the leading etiology of bronchiolitis and pneumonia in young children, resulting in greater than 100,000 hospitalizations annually [1,2]. It is a significant cause of morbidity and mortality in the United States and worldwide [3–5]. There is extreme variability in RSV disease severity, demonstrated by the fact that approximately half of all infants are infected with RSV during their first winter of life [6], yet only ~2% of infected infants are hospitalized with RSV [7]. Of hospitalized children, approximately 10% require mechanical ventilation, and of these, 5%–7% die of their disease [4,8–10]. Certain groups, including premature infants, nosocomially infected infants, infants with broncho-pulmonary dysplasia, compromised immunity or congenital heart disease, are at greatest risk for these severe and sometimes fatal infections [9,11–13]. However, the majority of patients hospitalized with RSV are previously healthy infants without known risk factors [2,3,10]. The mechanism by which RSV produces this wide range of disease severity in previously healthy infants is likely multi-factorial, but is largely unknown.

RSV infects the respiratory epithelium and causes disease through a direct cytopathic effect accompanied by a robust immuno-pathogenic response. Airway epithelial cells represent the first line of defense against pathogen challenge, producing antimicrobials, cytokines and chemokines upon attack to recruit a variety of immune cells, including antigen-presenting cells (dendritic cells, macrophages), granulocytes and lymphoctyes into the airway lumen [14]. These components of the immune system have been implicated in the pathogenesis of RSV, contributing to inflammation, airway edema and other manifestations of the disease [15,16]. Previous studies have shown that airway epithelial cells produce a wide range of inflammatory mediators, including the cytokines/chemokines IL-1α, IL-6, IL-8 and RANTES, in response to RSV infection [17,18]. IL-1α is secreted by activated macrophages and neutrophils, as well as epithelial cells, stimulating inflammation early after onset of infection. The cytokine IL-6 and chemokine RANTES are secreted by several cell types and are later acting mediators. IL-8 is also secreted by macrophages and epithelial cells and functions to recruit neutrophils-the predominant inflammatory cell type in the airway lumen of RSV-infected infants [19].

The factors leading to disease severity differences can be divided into host factors and viral factors [20]. Studies in humans and in animal models demonstrate that host immune mechanisms likely play a major role in the severity of disease. Multiple studies have demonstrated elevated cytokines and chemokines in the respiratory secretions [17,18,21–27] and blood of RSV-infected patients. Genetic variations in the host’s inflammatory immune response to RSV partially explain the wide range of disease severity seen in RSV infection [28–31]. Functional differences in infant’s capacity to produce pro-inflammatory responses have also been shown to correlate with human RSV disease severity [27]. In disease severe enough to result in infant death, few inflammatory cells are present in the lung parenchyma, yet there is rampant viral replication and infection of the airway epithelial cells, emphasizing the importance of studying viral interactions with respiratory epithelial cells [19]. What has been understudied is the role of factors intrinsic to the virus itself in contributing to these disease severity differences. We therefore held host factors constant by utilizing the A549 human lung epithelial cell line as a homogeneous *in vitro* model enabling the evaluation of purely viral-specific factors. This cell line represents an established lung epithelial model that has been shown to secrete inflammatory mediators upon RSV infection [32,33]. A549 cells were infected with clinical RSV isolates obtained from infants with different disease severities and assessed for cytokine/chemokine concentration and viral growth kinetics. Although *in vitro* models can never prove the mechanism through which a complex viral and host interaction produces disease, our purpose was not to define the mechanism but to investigate the general concept of whether or not intrinsic viral factors are associated with disease severity. This model eliminates host differences, through provides *in vitro* host standardization to examine disease-contributing differences inherent to the viruses themselves.

## Methods

The human experimentation guidelines of the United States Department of Health and Human Services and those of the participating institutions, including appropriate informed consent from parents or guardians, were followed in the conduct of this research. The authors verify that the study was conducted with University of Tennessee Health Science Center (UTHSC) and Methodist Le Bonheur Healthcare Institutional Review Board approval.

### Subjects

Primary RSV isolates were collected from clinical respiratory specimens of 206 RSV-infected infants identified over a two-year period from 2000–2002. The study was comprised of both hospitalized infants and infants treated at pediatric outpatient centers from Tennessee, Kentucky, Houston, Denver, Pittsburgh, and southern California. Disease severity was dichotomized into those infants sick enough to require hospitalization, severe RSV disease (inpatients), with all other infants considered to have mild RSV disease (outpatients). Selection criteria for study subjects are shown in Table 1. Infants were all previously healthy, less than 24 months of age, and had RSV detected in their respiratory secretions via ELISA, direct fluorescent antibody (DFA) or culture within 48 hours prior to enrollment. Subjects were also followed by telephone call and/or return visit to determine whether they were subsequently hospitalized. Any identified outpatients who were subsequently hospitalized within the following 14 days for RSV were also categorized as having severe disease.

### Virology

**Figure 1:**
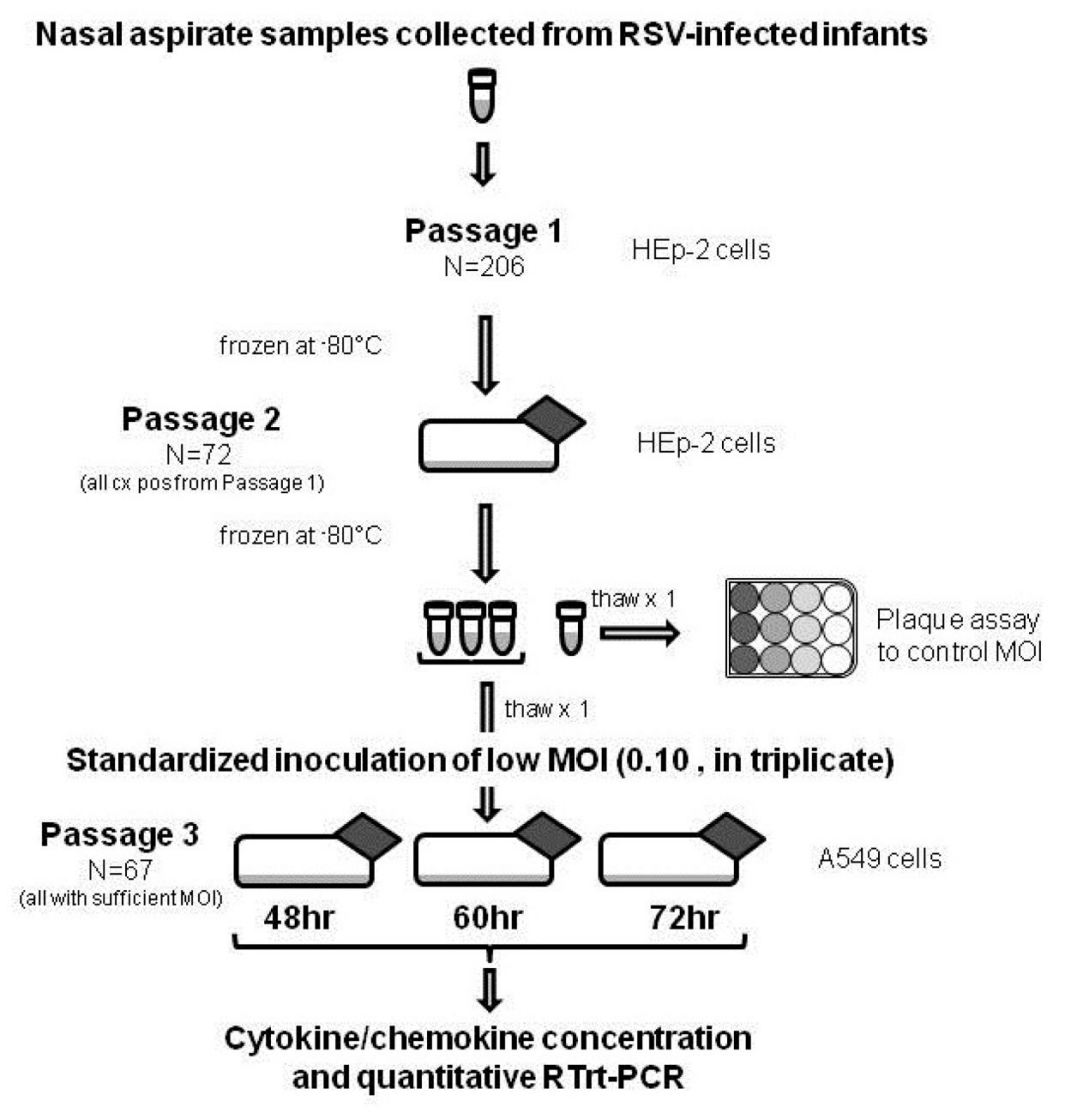
Study laboratory procedures and provenance of clinical isolates.

Respiratory specimens from all 206 patients were processed as outlined in Figure 1. They were initially cultured using the human larynx epidermoid carcinoma cell line HEp-2. Seventy-two clinical viral isolates grew in culture, of which 67 (93%) yielded sufficient virus for the study-designed multiplicity of infection (MOI) after a second HEp-2 cell passage. These viral isolates represented 38 infants with severe disease (including 5 ICU patients) and 29 infants with mild disease. Viral sub-grouping as determined by PCR of the virus N-gene [34] determined that 63 isolates were RSV A and four isolates were RSV B. For the study, a standardized and precise low MOI of 0.10 for all viral isolates was chosen for inoculation so as to mimic natural low-inoculum infection and to allow potential replication differences between RSV isolates to be observed. All 67 isolates were inoculated into human alveolar epithelial cell line A549 monolayers in triplicate and seeded with identical cell numbers and passage number, with all cultures at 80% confluence. Using 25 ml cell flasks, the viral inoculum and supernatant volume were kept standard at exactly 5 ml. Cultures were incubated at 37°C under 5% CO_2_ with agitation every 15 min for 1 hour. The cell cultures were then washed three times with HEPES and fresh growth medium added. Cultures were incubated at 37°C in 5% CO_2_ for 48 hours, 60 hours and 72 hours. At each time point, cultures were harvested by cell scraping, resuspended in growth medium, and spun for 10 minutes at 4°C. Supernatants were collected, aliquoted, snap-frozen immediately and stored at −80°C for future analysis. RSV A-long strain (ATCC# VR-26) was used as a positive control culture whilst sham viral cultures and irradiated RSV A-long (ATCC# VR-26) were used as negative control cultures. The provenance of the clinical isolates is shown schematically in Figure 1.

### Quantitative Assays

The concentrations of several cytokine/chemokine mediators were measured from A549 culture supernatants following RSV A-long infection (BIO SOURCE, Camarillo, CA). The cytokines/chemokines IL-1α, IL-6, IL-8 and RANTES were detectable in this *in vitro* model. The cytokines IFN-γ, TNF-α and IL-1β were not detected. Quantification of cytokine/chemokine concentrations were performed at a series of pre-determined time points so as to assess area under the curve (AUC) concentrations of cytokines contributing to the overall inflammatory response, and so as not to miss potential differences in timing of peak production between the cytokines/chemokines studied. Respiratory syncytial virus concentrations in culture supernatants were measured using quantitative real-time reverse transcriptase (RTrt)-PCR as previously described [34].

### Statistical Analysis

PRISM v.4.0a (GraphPad Software Inc., San Diego, CA) was used to analyze and graph the data. Standard descriptive statistics were used to analyze the data with Student’s t-tests and Mann-Whitney analyses used where appropriate depending on the Gaussian nature of the data. Statistical significance was set at 0.05 with 95% confidence intervals. Linear regression was used to correlate continuous variables. Graphical error bars represent standard error of the mean (SEM) throughout. Cytokine/chemokine concentrations were compared between A549 cultures infected with RSV clinical isolates collected from severely diseased infants with those from children with mild disease. Researchers were blinded as to disease severity dichotomy until data analysis.

## Results

### *In vitro* culture and cytokine/chemokine characteristics

**Figure 2:**
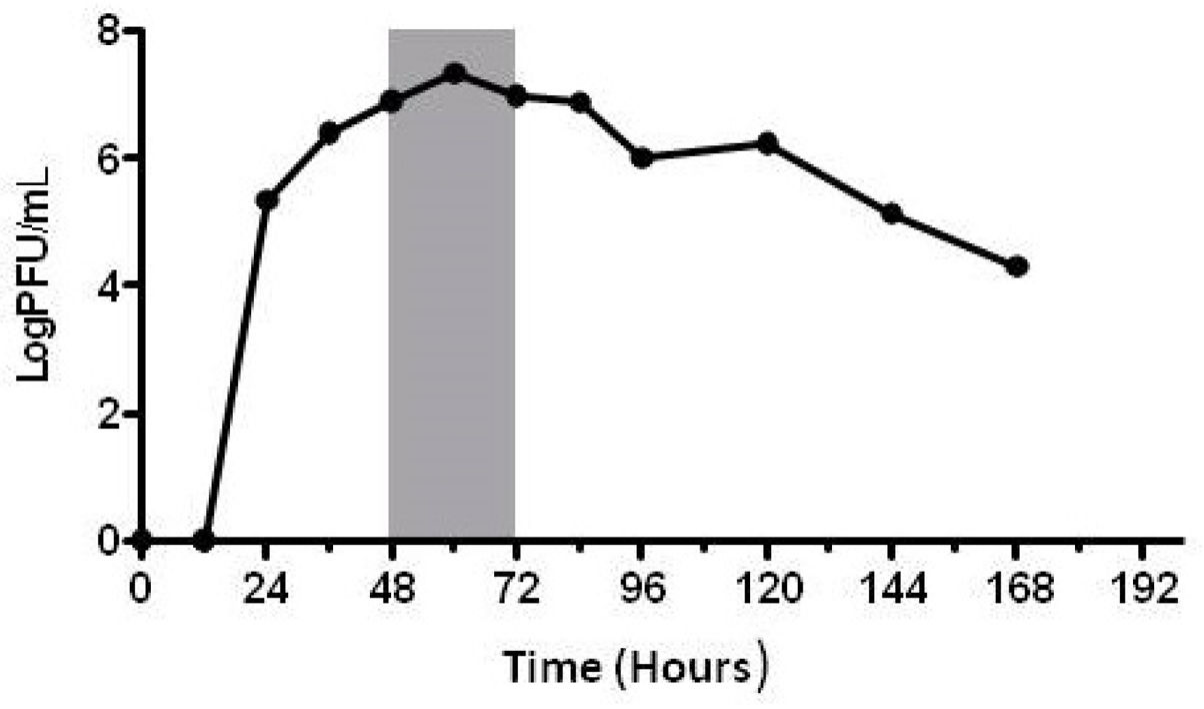
Representative in vitro growth curve for RSV. RSV-A long (ATCC# VR-26) grown in A549 lung epithelial cells under conditions used in experiments. Data points represent viral concentrations, measured in triplicate, from time of culture inoculation. Shaded area indicates the time frame in which each isolate was evaluated for viral growth characteristics and induced cytokine/chemokine concentrations.

**Figure 3:**
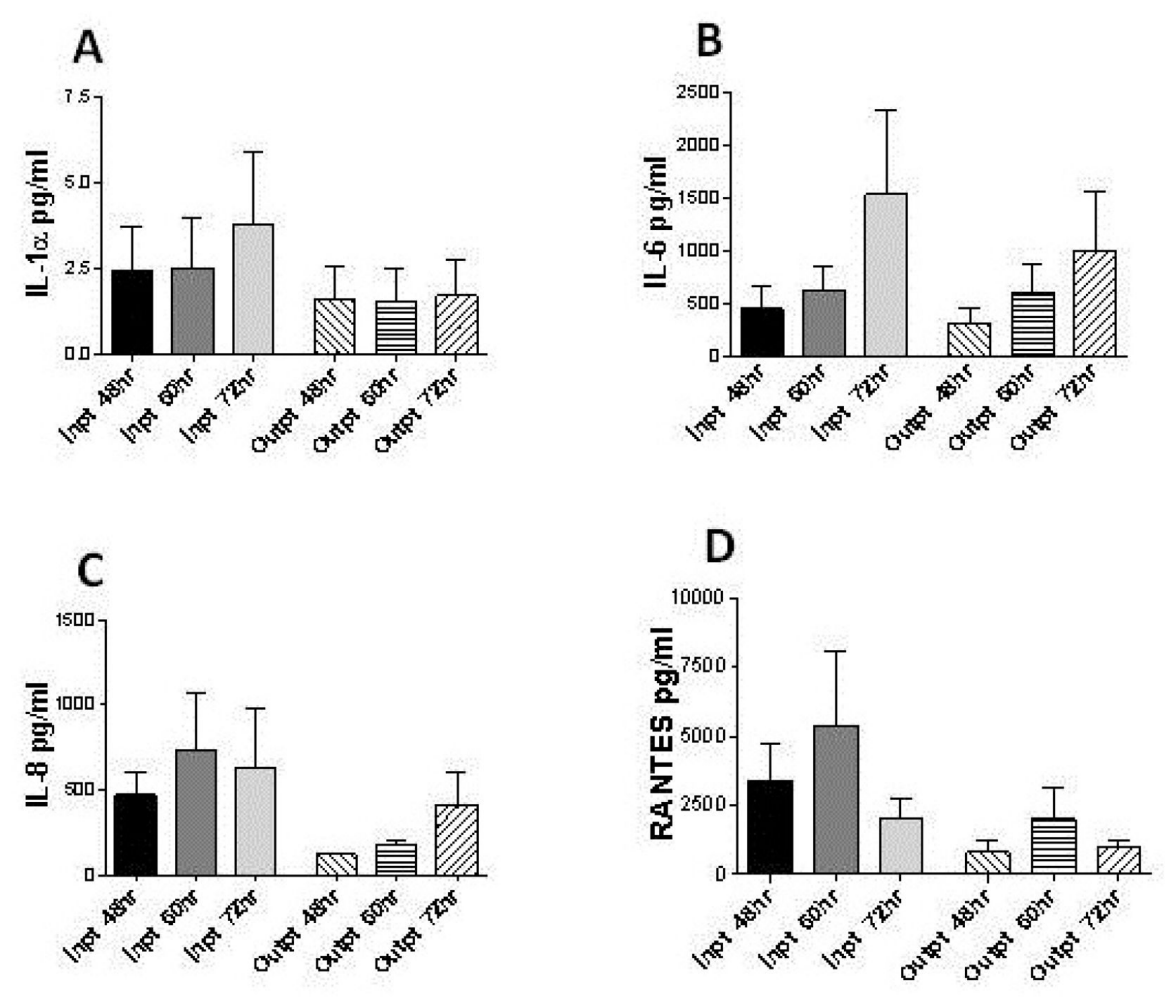
Concentration of cytokine/chemokines in A549 lung epithelial cell cultures over time following infection with RSV clinical isolates. Cytokine/chemokines *(A)* IL-1α, *(B)* IL-6, *(C)* IL-8 (p=0.028) *(D)* RANTES (p=0.019) were measured. MOI was held at 0.10. Inpt=RSV isolated from infants with severe RSV disease requiring hospitalization. Outpt=RSV isolated from infants with mild RSV disease. 48 hr, 60 hr and 72 hr represent time from point of culture inoculation. Error bars represent SEM of pooled viral isolate cytokine/chemokine concentrations for Inpt and Outpt over all time points from culture inoculation.

The human lung epithelial cell line A549 produced a predictable time course of RSV viral replication for the RSV A-long positive control culture (Figure 2). Cytokine/chemokine concentrations were reproducibly measured across A549 culture replicates, and were collectively found to significantly increase in culture supernatants during the time course of RSV infection studied (p<0.0001). RSV clinical isolate cultures induced significantly higher cytokine/chemokine concentrations than both negative control cultures, the sham viral culture (p<0.001) and the irradiated RSV culture (p<0.001) at all-time points. IL-8 concentrations increased significantly throughout the course of infection (p<0.001), with IL-1α and IL-6 demonstrating a trend in increasing concentration over time (p=0.936, p=0.191, respectively; Figure 3). As opposed to the cytokines assessed, the chemokine RANTES peaked at 60 hours (p=0.381) and then declined.

### Cytokine/chemokine concentration predicting disease severity

Inflammatory mediator production was greater in A549 cells infected with RSV clinical isolates collected from infants with severe disease compared to those collected from infants with mild disease. The isolates from infants with severe disease induced higher actual concentrations of each individual cytokine/chemokine examined at every time point evaluated (Figure 3). The greatest differences in mediator *in vitro* secretion between severe and mild disease RSV isolates cultured were observed for IL-8 and RANTES. At 48 hours and 60 hours, cultures of RSV isolates from infants with severe disease secreted four times greater IL-8 concentration than cultures exposed to isolates collected from infants with mild disease. Evaluating the AUC cytokine/chemokine concentrations (all evaluated time points), showed RSV isolates collected from infants with severe disease induced significantly higher AUCIL-8 secretion in infected cultures than mild disease isolates (p=0.028). Similarly, at 48 hours and 60 hours post-infection RANTES concentrations were 3 to 4-fold higher, respectively, in the A549 cultures infected with severe disease viral isolates. The clinical isolates from more severely ill infants induced greater AUCRANTES concentrations that were significantly higher than concentrations induced by isolates from infants with mild disease (p=0.019; Figure 3).

There were no observed significant differences between the cytokine/chemokine concentrations produced by A549 cultures exposed to RSV subgroup A isolates compared to those exposed to RSV subgroup B isolates collected. However, the number of RSV-B isolates evaluated was small (N=4 of 67).

### *In vitro* viral growth kinetics

**Figure 4:**
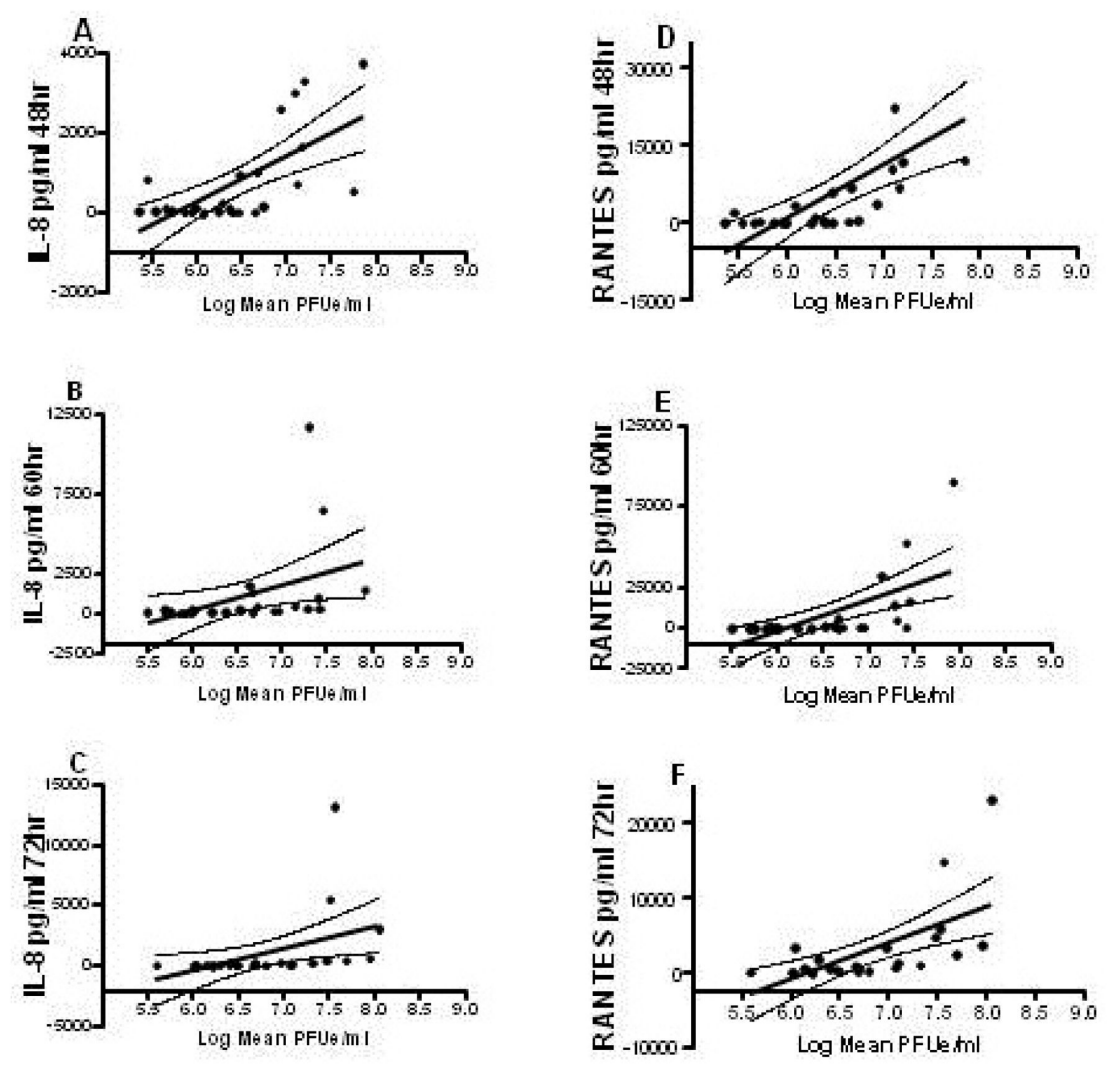
Relationship between IL-8 and RANTES concentration and quantity of virus in A549 lung epithelial cell cultures over time following infection with RSV clinical isolates. Both IL-8 (p<0.001; r=0.439 48 hr; r=0.189 60 hr; r=0.194 72 hr; Panel A–C) and RANTES (p<0.001; r=0.442 48 hr; r=0.359 60 hr; r=0.331 72 hr; Panel D–F) cytokine/chemokine concentration significantly correlated with RSV quantity at all-time points. MOI was held at 0.10.

To determine whether the observed cytokine and chemokine differences were related to different rates of viral growth, concentrations of RSV were measured from aliquots of the A549 cell culture supernatants used for the cytokine/chemokine assessment. Thirty-eight of the RSV clinical isolates were tested for viral concentrations at all-time points. Cytokine/chemokine concentrations correlated directly with RSV concentrations at all-time points for IL-1α, IL-8 and RANTES. At all-time points, IL-8 and RANTES concentrations correlated very closely with RSV concentrations, the correlation coefficients being greatest at early time points compared to the other cytokines (p<0001; Figure 4). IL-1α concentration significantly correlated with RSV concentration at 72 hours culture (p=0.019). IL-6 concentrations did not correlate with RSV concentrations for any time point assessed. The quantity of RSV in the culture supernatants did not correlate with disease severity; isolates collected from both infants with severe and mild disease induced a similar viral load.

## Discussion

We have shown that *in vitro* induction of the inflammatory mediators IL-1α, IL-6, IL-8 and RANTES is higher in cultures infected with RSV clinical isolates collected from infants with severe disease than those collected from infants with mild disease, at multiple time points. These experiments were designed to test the general hypothesis that viral-specific characteristics, borne by different circulating clinical strains of RSV, are responsible for some of the differential clinical disease severities observed in infants. Low-passage clinical isolates of RSV were found to differ in their ability to induce secretion of these cytokine/chemokines in cultures of an infected human lung epithelial cell line (A549). Most striking was the increased concentration of IL-8 and RANTES in severe disease RSV isolates compared to that induced by isolates collected from infants with mild disease (p=0.028 and 0.019 for IL-8 and RANTES, respectively). These results fit well with the recent observations that infants with greater RSV disease severity produce higher elevations of IL-8 and RANTES in their respiratory secretions [18,21,23,26,35]. Elevated IL-8 and RANTES has also been observed in the respiratory secretions of adult volunteers participating in a recently developed experimental infection model [22]. Furthermore, in this same experimental infection model, higher RSV loads were significantly correlated with elevations in the AUC’s of both these cytokines/chemokines [22].

Recent studies have shown a significant correlation between RSV viral load and severity of disease both in naturally infected infants [8,36] and adult volunteers participating in the experimental infection model [22]. *In vitro* viral replication kinetics observed for RSV A-long virus cultured in A549, although of a higher log PFU/ml, resemble the growth curve of the RSV A in experimentally infected adults [22]. The *in vitro* viral growth kinetics of the isolates evaluated in our study appears to be driving the *in vitro* cytokine/chemokine differences. These viral-specific replication kinetics and cytopathogenic effects should be further evaluated.

It is likely that the determinants of the quantity of these cytokine/chemokines induced in infants result, in part, from host immuno-genetic differences. However, our data suggest the possibility that additional inherent viral strain-specific factors may also play a role. These putative characteristics could be viral genetic-determined improvements in viral growth kinetics *in vivo*, different degrees of NS-1/NS-2 protein suppression of IFN, differences in the ability to produce different cytopathology including fusion capacity or expression differences, or other viral immune-stimulatory factors such as a variable ability of the RSV to modulate host inflammatory mediators through multiple mechanisms, including molecular mimicry and translational activation [37,38]. A prior study supported our findings, in that infection of A549 by four RSV clinical isolates resulted in the differential concentration of cytokine/chemokine induced, including that of IL-6, IL-8 and RANTES [39]. However, unlike our study, the laboratory strain A-long in the previous study was far more virulent than any clinical isolate examined. An expanded list of evaluated cytokines/chemokines/proteins might be evaluated in future studies to cast a broader net to determine which specific host factors are being differentially affected by the different RSV clinical isolates. Such an expanded list might utilize a proteomic approach.

Our studies are not without limitations. Our list of cytokine/chemokines evaluated was limited by those cytoproteins detectable in our system. Another limitation occurred during selection of the viral isolates for the studies. The MOI had to be finely controlled at a low level to more closely resemble natural infection. Therefore, clinical isolates that grew poorly could not be included. Thus, our studies may have evaluated only a truncated range of intrinsic viral strain-specific factors. Also, though unarguably clinically relevant, the simple dichotomized definition of disease severity employed may have been limiting.

The primary aim of our study was to determine whether viral-specific factors were at least partially responsible for human disease severity differences. Therefore, a cell line that optimized homogeneity was necessary to remove any host-specific differences. Primary bronchiolar epithelial cells differ with respect to their origin, passage number, and growth characteristics; and because of this heterogeneity, were therefore not utilized. An evaluation of which specific viral strain-specific factors produced which specific cellular effects might be better evaluated within primary bronchiolar epithelial cells. Furthermore, the immune response is dependent upon a complex interaction of antigen-presenting helper cells and effector immune cells. To fully evaluate whether different RSV clinical strains produce different degrees of immune stimulation, dendritic cells or macrophages must be included within the model. *Ex vivo* human (preferably infant) primary bronchiolar cells or *in vitro* respiratory tissue reconstruction models may represent additional evaluation strategies, but they would have to be complex mixed cell culture models including epithelial and immune cells. Such *in vitro* models are not common, are difficult to reproduce and vary from donor to donor. Alternatively, a standardized animal model could be infected with various clinical strains, but the costs and logistics of such an evaluation may be prohibitive. Furthermore, the pathophysiology of RSV in small animal models is not parallel to that of humans. The only animal model currently available that closely mirrors the pathology of human infant RSV infection is that of newborn lambs [40] and possibly baboons [41]. Lambs are infected naturally with ovine or bovine RSV, and can be experimentally infected with human RSV. A recent study showed that this lamb RSV infection model is associated with an immune inflammatory response involving several cytokine/chemokine mediators [40] including those we have also studied here.

In conclusion, we have observed that different clinical isolates of RSV induce different concentrations of important inflammatory mediators during infection of human lung epithelial cells. Low-passage RSV strains isolated from previously healthy infants with severe RSV disease induce greater amounts of these important cytokine/chemokines compared to identically prepared low-passage RSV strains isolated from infants with mild disease. Some of the observed differences in RSV disease severity among individuals appear to be explained by viral strain-specific factors, especially in their ability to induce IL-8 and RANTES secretion, in part mediated by different intrinsic viral growth kinetics. Although further studies are needed to identify which viral-specific factors are directly responsible for producing greater disease severity and which are simply associated with disease severity differences, this study supports the general concept that RSV strain-specific characteristics play a role in the pathogenesis of RSV infection. Both viral and human host factors contribute to disease severity differences in humans.

## Figures and Tables

**Table 1:** RSV-infected infant study selection criteria.

Inclusion	Exclusion
Less than 2 years of age at sample collection	Has received RSV-Ig or palivizumab
>35 weeks gestational age at birth	Has a known immunodeficiency disorder
Has a parent or guardian who gives consent	Has clinically significant congenital heart disease
	Has a diagnosis of chronic lung disease including broncho-pulmonary dysplasia

## Abbreviations

Inpt: Inpatient
Outpt: Outpatient
MOI: Multiplicity of Infection
ELISA: Enzyme-Linked Immunosorbent Assay
DFA: Direct Fluorescent Antibody
PCR: Polymerase Chain Reaction
RTrt-PCR: Quantitative Real-Time Reverse Transcriptase
AUC: Area Under the Curve
SEM: Standard Error of the Mean
